# 4-*tert*-Butyl-5-(1*H*-1,2,4-triazol-1-yl)thia­zol-2-amine

**DOI:** 10.1107/S1600536809029912

**Published:** 2009-07-31

**Authors:** An-Yun Xie, Jiao Ye, Zhi Qin, Ai-Xi Hu

**Affiliations:** a Hunan Warrant Pharmaceutical Co. Ltd, Changsha 410329, People’s Republic of China; b College of Chemistry and Chemical Engineering, Hunan University, Changsha 410082, People’s Republic of China

## Abstract

The dihedral angle between the triazole ring and the thia­zole ring in the title compound, C_9_H_13_N_5_S, is 64.35 (7)°. The crystal structure is stabilized by inter­molecular N—H⋯N hydrogen bonds, which link the mol­ecules into a two-dimensional network.

## Related literature

For background and related structures, see: Zhou *et al.* (2007 ▶); Shao *et al.* (2008 ▶).
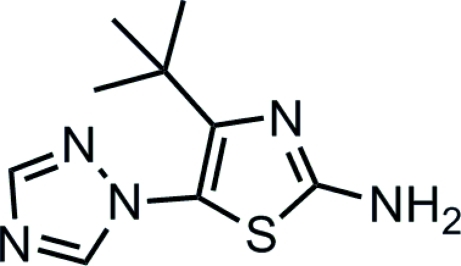


## Experimental

### 

#### Crystal data

C_9_H_13_N_5_S
*M* *_r_* = 223.30
Monoclinic, 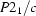

*a* = 7.7487 (4) Å
*b* = 14.2240 (8) Å
*c* = 10.2697 (5) Å
β = 91.452 (1)°
*V* = 1131.54 (10) Å^3^
*Z* = 4
Mo *K*α radiation
μ = 0.26 mm^−1^
*T* = 173 K
0.47 × 0.43 × 0.37 mm

#### Data collection

Bruker SMART 1000 CCD diffractometer
Absorption correction: multi-scan (*SADABS*; Sheldrick, 2004 ▶) *T*_min_ = 0.887, *T*_max_ = 0.909
6167 measured reflections
2463 independent reflections
2187 reflections with *I* > 2σ(*I*)
*R*_int_ = 0.016

#### Refinement

*R*[*F* ^2^ > 2σ(*F* ^2^)] = 0.031
*wR*(*F* ^2^) = 0.089
*S* = 1.07
2463 reflections
139 parameters
H-atom parameters constrained
Δρ_max_ = 0.24 e Å^−3^
Δρ_min_ = −0.31 e Å^−3^

### 

Data collection: *SMART* (Bruker, 2001 ▶); cell refinement: *SAINT-Plus* (Bruker, 2003 ▶); data reduction: *SAINT-Plus*; program(s) used to solve structure: *SHELXTL* (Sheldrick, 2008 ▶); program(s) used to refine structure: *SHELXTL*; molecular graphics: *SHELXTL*; software used to prepare material for publication: *SHELXTL*.

## Supplementary Material

Crystal structure: contains datablocks I, global. DOI: 10.1107/S1600536809029912/bt5018sup1.cif

Structure factors: contains datablocks I. DOI: 10.1107/S1600536809029912/bt5018Isup2.hkl

Additional supplementary materials:  crystallographic information; 3D view; checkCIF report

## Figures and Tables

**Table 1:** Hydrogen-bond geometry (Å, °)

*D*—H⋯*A*	*D*—H	H⋯*A*	*D*⋯*A*	*D*—H⋯*A*
N2—H2*B*⋯N5^i^	0.88	2.22	3.0049 (16)	148
N2—H2*A*⋯N1^ii^	0.88	2.17	3.0392 (14)	168

Symmetry codes: (i) 
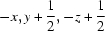
; (ii) 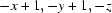.

**Fig. 1.**
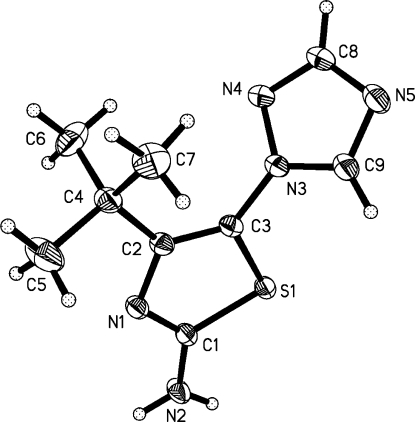
Molecular structure showing 50% probability displacement ellipsoids.

**Fig. 2.**
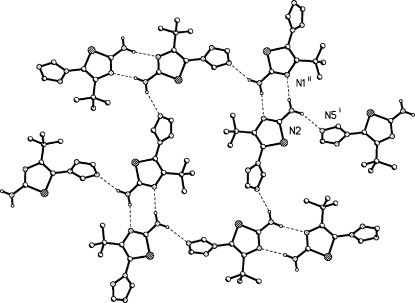
A packing diagram for the title compound showing intermolecular hydrogen bonds as dashed lines. H atoms bonded to C omitted for clarity.

## supplementary crystallographic information

### Comment

Thiazole derivatives and 1,2,4-triazole derivatives have been found to be active compounds with diversely biological activities (Zhou *et al.*, 2007; Shao *et al.*, 2008). We herein report the synthesis and structures of the title compound, 4-*tert*-butyl-5-(1*H*-1,2,4-triazol-1-yl)thiazol-2-amine, which was incorporated 1*H*-1,2,4-triazole units into the novel thialoyl urea compounds in order to find novel leading compounds with potential anticancer activities.

The title compound (Fig. 1),   contains two planar subunits: the thiazole ring and the triazole ring. The dihedral angles between them is 64.35 (7). The crystal structure (Fig. 2) is stabilized by intermolecular hydrogen bonds between the amino group and the nitrogen atoms of the thiazol ring and the triazole ring of the neighbouring molecules.

For related structures, see: Zhou *et al.* (2007), Shao *et al.* (2008).

### Experimental

1-bromo-3,3-dimethyl-1-(1*H*-1,2,4-triazol-1-yl)butan-2-one (0.01 mol) were refluxed with thiourea (0.01 mol) in ethanol (45 ml) for 1.5 h (monitored by TLC). Then the pH of the mixture was adjusted to 9 with ammonia and filtered to obtain white solid, 4-*tert*-butyl-5-(1*H*-1,2,4-triazol-1-yl) thiazol-2-amine. Yield 85.5%, m.p. 451 K.

Crystals suitable for X-ray structure determination were obtained by slow evaporation of an ethanol solution at room temperature.

### Refinement

The H-atoms were positioned geometrically, with C—H = 0.98Å for methyl, C—H = 0.95Å for the triazole ring, C—H = 0.88Å for the amino group and refined as riding with *U*_iso_(H) = 1.2*U*_eq_(C,N) or 1.5*U*_eq_(C_methyl_).

### Crystal data

**Table:**

C_9_H_13_N_5_S	*F*(000) = 472
*M**_r_* = 223.30	*D*_x_ = 1.311 Mg m^−^^3^
Monoclinic, *P*2_1_/*c*	Melting point: 451 K
Hall symbol: -P 2ybc	Mo *K*α radiation, λ = 0.71073 Å
*a* = 7.7487 (4) Å	Cell parameters from 4587 reflections
*b* = 14.2240 (8) Å	θ = 2.5–27.1°
*c* = 10.2697 (5) Å	µ = 0.26 mm^−^^1^
β = 91.452 (1)°	*T* = 173 K
*V* = 1131.54 (10) Å^3^	Block, colorless
*Z* = 4	0.47 × 0.43 × 0.37 mm

### Data collection

**Table:**

Bruker SMART 1000 CCD diffractometer	2463 independent reflections
Radiation source: fine-focus sealed tube	2187 reflections with *I* > 2σ(*I*)
graphite	*R*_int_ = 0.016
ω scans	θ_max_ = 27.1°, θ_min_ = 2.5°
Absorption correction: multi-scan (*SADABS*; Sheldrick, 2004)	*h* = −9→4
*T*_min_ = 0.887, *T*_max_ = 0.909	*k* = −18→18
6167 measured reflections	*l* = −13→13

### Refinement

**Table:**

Refinement on *F*^2^	Primary atom site location: structure-invariant direct methods
Least-squares matrix: full	Secondary atom site location: difference Fourier map
*R*[*F*^2^ > 2σ(*F*^2^)] = 0.031	Hydrogen site location: inferred from neighbouring sites
*wR*(*F*^2^) = 0.089	H-atom parameters constrained
*S* = 1.07	*w* = 1/[σ^2^(*F*_o_^2^) + (0.0539*P*)^2^ + 0.2785*P*] where *P* = (*F*_o_^2^ + 2*F*_c_^2^)/3
2463 reflections	(Δ/σ)_max_ < 0.001
139 parameters	Δρ_max_ = 0.24 e Å^−^^3^
0 restraints	Δρ_min_ = −0.31 e Å^−^^3^

### Special details

**Table:**

Experimental. 1H NMR (CDCl_3_, 400 MHz) δ: 1.11 (s, 9H, (CH_3_)_3_), 4.38 (s, 2H, NH_2_), 8.06 (s, 1H, C_2_H_2_N_3_ 3-H), 8.23 (s, 1H, C_2_H_2_N_3_ 5-H).
Geometry. All e.s.d.'s (except the e.s.d. in the dihedral angle between two l.s. planes) are estimated using the full covariance matrix. The cell e.s.d.'s are taken into account individually in the estimation of e.s.d.'s in distances, angles and torsion angles; correlations between e.s.d.'s in cell parameters are only used when they are defined by crystal symmetry. An approximate (isotropic) treatment of cell e.s.d.'s is used for estimating e.s.d.'s involving l.s. planes.
Refinement. Refinement of *F*^2^ against ALL reflections. The weighted *R*-factor *wR* and goodness of fit *S* are based on *F*^2^, conventional *R*-factors *R* are based on *F*, with *F* set to zero for negative *F*^2^. The threshold expression of *F*^2^ > σ(*F*^2^) is used only for calculating *R*-factors(gt) *etc*. and is not relevant to the choice of reflections for refinement. *R*-factors based on *F*^2^ are statistically about twice as large as those based on *F*, and *R*- factors based on ALL data will be even larger.

### Fractional atomic coordinates and isotropic or equivalent isotropic displacement parameters (Å^2^)

**Table:**

	*x*	*y*	*z*	*U*_iso_*/*U*_eq_	
S1	0.10734 (4)	0.36412 (2)	0.12795 (3)	0.02144 (11)	
C1	0.28446 (15)	0.42187 (8)	0.06301 (12)	0.0208 (2)	
C2	0.38251 (15)	0.27348 (8)	0.06564 (11)	0.0194 (2)	
C3	0.22378 (16)	0.25969 (8)	0.11582 (11)	0.0200 (2)	
C4	0.52048 (16)	0.19969 (9)	0.03942 (13)	0.0248 (3)	
C5	0.69774 (19)	0.24700 (11)	0.0350 (2)	0.0490 (5)	
H5A	0.6994	0.2911	−0.0382	0.073*	
H5B	0.7869	0.1990	0.0239	0.073*	
H5C	0.7203	0.2811	0.1166	0.073*	
C6	0.4799 (2)	0.15274 (11)	−0.09224 (15)	0.0392 (4)	
H6A	0.4739	0.2008	−0.1606	0.059*	
H6B	0.3689	0.1200	−0.0885	0.059*	
H6C	0.5711	0.1075	−0.1117	0.059*	
C7	0.5268 (2)	0.12361 (10)	0.14558 (16)	0.0368 (3)	
H7A	0.6247	0.0816	0.1312	0.055*	
H7B	0.4193	0.0873	0.1420	0.055*	
H7C	0.5404	0.1534	0.2313	0.055*	
C8	0.01671 (17)	0.04703 (9)	0.15964 (13)	0.0267 (3)	
H8	−0.0313	−0.0113	0.1318	0.032*	
C9	0.09146 (17)	0.15830 (9)	0.28121 (12)	0.0267 (3)	
H9	0.1101	0.1985	0.3541	0.032*	
N1	0.41461 (13)	0.36685 (7)	0.03390 (10)	0.0210 (2)	
N2	0.27815 (14)	0.51513 (7)	0.04483 (12)	0.0285 (3)	
H2A	0.3661	0.5445	0.0108	0.034*	
H2B	0.1860	0.5469	0.0670	0.034*	
N3	0.14376 (13)	0.17666 (7)	0.16036 (10)	0.0206 (2)	
N4	0.09397 (14)	0.10478 (8)	0.07907 (10)	0.0254 (2)	
N5	0.01069 (15)	0.07679 (8)	0.28504 (11)	0.0299 (3)	

### Atomic displacement parameters (Å^2^)

**Table:**

	*U*^11^	*U*^22^	*U*^33^	*U*^12^	*U*^13^	*U*^23^
S1	0.01973 (17)	0.01734 (17)	0.02758 (18)	−0.00120 (10)	0.00713 (12)	−0.00005 (10)
C1	0.0194 (6)	0.0182 (6)	0.0251 (6)	−0.0023 (4)	0.0045 (4)	0.0005 (4)
C2	0.0198 (6)	0.0165 (5)	0.0221 (5)	−0.0009 (4)	0.0003 (4)	0.0011 (4)
C3	0.0218 (6)	0.0155 (5)	0.0229 (5)	−0.0016 (4)	0.0023 (4)	0.0011 (4)
C4	0.0203 (6)	0.0167 (5)	0.0374 (7)	0.0019 (5)	0.0031 (5)	0.0018 (5)
C5	0.0202 (7)	0.0241 (7)	0.1029 (15)	0.0024 (6)	0.0083 (8)	0.0026 (8)
C6	0.0457 (9)	0.0356 (8)	0.0366 (8)	0.0134 (7)	0.0078 (6)	−0.0056 (6)
C7	0.0380 (8)	0.0275 (7)	0.0447 (8)	0.0103 (6)	−0.0008 (6)	0.0090 (6)
C8	0.0292 (6)	0.0194 (6)	0.0317 (6)	−0.0069 (5)	0.0017 (5)	0.0008 (5)
C9	0.0316 (7)	0.0254 (6)	0.0234 (6)	−0.0073 (5)	0.0042 (5)	0.0014 (5)
N1	0.0187 (5)	0.0164 (5)	0.0282 (5)	−0.0009 (4)	0.0043 (4)	0.0015 (4)
N2	0.0242 (5)	0.0149 (5)	0.0471 (7)	0.0002 (4)	0.0136 (5)	0.0022 (5)
N3	0.0222 (5)	0.0173 (5)	0.0223 (5)	−0.0038 (4)	0.0016 (4)	0.0008 (4)
N4	0.0294 (6)	0.0197 (5)	0.0271 (5)	−0.0062 (4)	0.0031 (4)	−0.0029 (4)
N5	0.0328 (6)	0.0277 (6)	0.0293 (6)	−0.0095 (5)	0.0052 (5)	0.0045 (4)

### Geometric parameters (Å, °)

**Table:**

S1—C3	1.7441 (12)	C6—H6B	0.9800
S1—C1	1.7464 (12)	C6—H6C	0.9800
C1—N1	1.3169 (16)	C7—H7A	0.9800
C1—N2	1.3403 (16)	C7—H7B	0.9800
C2—C3	1.3598 (17)	C7—H7C	0.9800
C2—N1	1.3914 (14)	C8—N4	1.3202 (16)
C2—C4	1.5270 (16)	C8—N5	1.3575 (18)
C3—N3	1.4153 (15)	C8—H8	0.9500
C4—C5	1.5312 (19)	C9—N5	1.3187 (17)
C4—C6	1.533 (2)	C9—N3	1.3410 (16)
C4—C7	1.5360 (18)	C9—H9	0.9500
C5—H5A	0.9800	N2—H2A	0.8800
C5—H5B	0.9800	N2—H2B	0.8800
C5—H5C	0.9800	N3—N4	1.3692 (14)
C6—H6A	0.9800		
			
C3—S1—C1	87.73 (6)	C4—C6—H6C	109.5
N1—C1—N2	125.59 (11)	H6A—C6—H6C	109.5
N1—C1—S1	114.89 (9)	H6B—C6—H6C	109.5
N2—C1—S1	119.50 (9)	C4—C7—H7A	109.5
C3—C2—N1	113.28 (10)	C4—C7—H7B	109.5
C3—C2—C4	127.71 (10)	H7A—C7—H7B	109.5
N1—C2—C4	118.99 (10)	C4—C7—H7C	109.5
C2—C3—N3	130.61 (11)	H7A—C7—H7C	109.5
C2—C3—S1	112.26 (9)	H7B—C7—H7C	109.5
N3—C3—S1	117.12 (9)	N4—C8—N5	115.32 (11)
C2—C4—C5	109.61 (10)	N4—C8—H8	122.3
C2—C4—C6	109.04 (10)	N5—C8—H8	122.3
C5—C4—C6	109.23 (13)	N5—C9—N3	110.70 (11)
C2—C4—C7	111.65 (11)	N5—C9—H9	124.6
C5—C4—C7	108.55 (12)	N3—C9—H9	124.6
C6—C4—C7	108.73 (11)	C1—N1—C2	111.81 (10)
C4—C5—H5A	109.5	C1—N2—H2A	120.0
C4—C5—H5B	109.5	C1—N2—H2B	120.0
H5A—C5—H5B	109.5	H2A—N2—H2B	120.0
C4—C5—H5C	109.5	C9—N3—N4	109.38 (10)
H5A—C5—H5C	109.5	C9—N3—C3	127.35 (10)
H5B—C5—H5C	109.5	N4—N3—C3	123.03 (10)
C4—C6—H6A	109.5	C8—N4—N3	101.98 (10)
C4—C6—H6B	109.5	C9—N5—C8	102.60 (11)
H6A—C6—H6B	109.5		
			
C3—S1—C1—N1	1.03 (10)	S1—C1—N1—C2	−1.71 (13)
C3—S1—C1—N2	179.99 (11)	C3—C2—N1—C1	1.65 (15)
N1—C2—C3—N3	−179.45 (11)	C4—C2—N1—C1	−179.57 (10)
C4—C2—C3—N3	1.9 (2)	N5—C9—N3—N4	0.78 (15)
N1—C2—C3—S1	−0.87 (13)	N5—C9—N3—C3	175.29 (12)
C4—C2—C3—S1	−179.52 (10)	C2—C3—N3—C9	117.79 (16)
C1—S1—C3—C2	−0.05 (9)	S1—C3—N3—C9	−60.72 (15)
C1—S1—C3—N3	178.73 (10)	C2—C3—N3—N4	−68.38 (18)
C3—C2—C4—C5	−157.79 (14)	S1—C3—N3—N4	113.10 (11)
N1—C2—C4—C5	23.63 (17)	N5—C8—N4—N3	1.00 (15)
C3—C2—C4—C6	82.69 (16)	C9—N3—N4—C8	−1.03 (14)
N1—C2—C4—C6	−95.90 (13)	C3—N3—N4—C8	−175.83 (11)
C3—C2—C4—C7	−37.48 (17)	N3—C9—N5—C8	−0.16 (15)
N1—C2—C4—C7	143.94 (12)	N4—C8—N5—C9	−0.56 (16)
N2—C1—N1—C2	179.41 (12)		

### Hydrogen-bond geometry (Å, °)

**Table:**

*D*—H···*A*	*D*—H	H···*A*	*D*···*A*	*D*—H···*A*
N2—H2B···N5^i^	0.88	2.22	3.0049 (16)	148
N2—H2A···N1^ii^	0.88	2.17	3.0392 (14)	168

Symmetry codes: (i) −*x*, *y*+1/2, −*z*+1/2; (ii) −*x*+1, −*y*+1, −*z*.
